# Brucellosis and chlamydiosis seroprevalence in goats at livestock–wildlife interface areas of Zimbabwe

**DOI:** 10.4102/ojvr.v86i1.1670

**Published:** 2019-08-22

**Authors:** Solomon Bhandi, Davies M. Pfukenyi, Gift Matope, Absolom Murondoti, Musavengana Tivapasi, Masimba Ndengu, Massimo Scacchia, Barbara Bonfini, Michel de Garine-Wichatitsky

**Affiliations:** 1 Department of Clinical Veterinary Studies, Faculty of Veterinary Science, University of Zimbabwe, Harare, Zimbabwe; 2 Research Platform Production and Conservation in Partnership, Harare, Zimbabwe; 3 Department of Paraclinical Veterinary Studies, Faculty of Veterinary Science, University of Zimbabwe, Harare, Zimbabwe; 4 The Experimental Zooprophylactic Institute of Abruzzo and Molise, Teramo, Italy; 5 Faculty of Veterinary Medicine, Kasetsart University, Bangkok, Thailand

**Keywords:** brucellosis, chlamydiosis, goats, interface, seroprevalence, Zimbabwe

## Abstract

In Zimbabwe, there have been no chlamydiosis and limited brucellosis studies in goats. This study was conducted to determine the seroprevalence and risk factors of the two diseases in goats at three different livestock–wildlife interface areas: porous, non-porous and non-interface in the south-eastern lowveld of Zimbabwe. Collected sera (*n* = 563) were tested for Brucella antibodies using the Rose Bengal plate test (RBPT) and the complement fixation test (CFT); and for *Chlamydia abortus* antibodies using the CFT. All tested goats were negative for Brucella antibodies. Overall, chlamydial seroprevalence was 22%. The porous [*c*^2^ = 9.6, odds ratio (OR) = 2.6, *p* = 0.002] and non-porous (*c*^2^ = 37.5, OR = 5.8, *p* < 0.00001) interfaces were approximately three and six times more likely to be chlamydial seropositive than the non-interface area, respectively. Chlamydial seroprevalence was not associated with sex (*c*^2^ = 0.5, OR = 1.2, *p* = 0.5), abortion history in female goats (*c*^2^ = 0.7, OR = 1.3, *p* = 0.4), keeping goats with cattle (*c*^2^ = 0.2, OR = 1.5, *p* = 0.7) or flock size (*c*^2^ = 0.03, OR = 1.4, *p* = 0.9). Our study provides the first serological evidence of chlamydiosis in goats in Zimbabwe and the results suggest that proximity to wildlife is associated with increased chlamydial seropositivity. Further studies are required to determine the role of chlamydial infection on goat reproductive failure and that of wildlife on *C. abortus* transmission to domestic ruminants.

## Introduction

Goat brucellosis is a chronic infectious disease caused by the gram-negative cocci-bacillus *Brucella melitensis* (Rossetti et al. 2017). In sub-Saharan Africa, goat *Brucella* spp. isolation studies are limited as indicated in the exhaustive review by Ducrotoy et al. (2017). This review showed that *B. melitensis* was isolated infrequently in goats in sub-Saharan African countries such as Kenya, Nigeria, South Africa and Zimbabwe. Literature on *Brucella abortus* isolation in goats in sub-Saharan Africa is also scarce with one report in Nigeria (Falade 1981). Similarly, *Brucella suis* isolations from goats have seldom been reported (Bhaskar Rao, Madhubala & Rumakrishna Rao 1998), and they have not been further documented (Rossetti et al. 2017). Despite being under control in most industrialised countries, goat brucellosis remains a major problem in the Mediterranean region, the Middle East, Central and Southeastern Asia, sub-Saharan Africa and parts of Latin America (FAO 2010). In Africa, it is endemic in countries in the Mediterranean region and the eastern part of the continent (Rossetti et al. 2017). As brucellosis is considered a neglected disease that significantly affects countries where resources are limited, there are a few studies that measure its economic impact in small ruminants (Rossetti et al. 2017). Despite still remaining a significant burden on goat and human health in the developing world, there is a lack of useful epidemiological data for aiding the design of appropriate control, prevention and eradication strategies. As compared to cattle, there is limited information on goat brucellosis in sub-Saharan Africa (Ducrotoy et al. 2017). Most recent goat seroprevalence studies have been reported in Ethiopia (Asmare et al. 2013; Megersa et al. 2012; Teklue et al. 2013) and South Africa (Simpson et al. 2018).

Except for Australia and New Zealand, *Chlamydia abortus* is the major cause of abortion in sheep and goats in small ruminant-rearing regions of the world (Rodolakis & Laroucau 2015). Most cases occur in management systems where animals are closely congregated during the peri-parturient period (Aitken & Longbottom 2007). Abortion, in most cases, is the only clinical evidence of *C. abortus* infection in goats, but concurrent respiratory tract disease, polyarthritis, epididymitis, conjunctivitis and retained placenta have been reported (Matthews 1999). In southern Africa, goat chlamydiosis has been reported in Namibia (Appel, Huebschle & Krauss 1989; Samkange et al. 2010) and South Africa (Musuka et al. 2001).

Rural communities living on the edge of the Great Limpopo Transfrontier Conservation Area rely mostly on livestock production for their livelihoods (Caron et al. 2013; De Garine-Wichatitsky et al. 2013; Gadaga et al. 2016; Ndengu et al. 2017a). The boundary fence that was erected to separate wildlife and livestock in the area, as part of the foot-and-mouth disease control, has been destroyed; permitting varying degrees of livestock and wildlife contacts (De Garine-Wichatitsky et al. 2013). Thus, in these areas, humans, domesticated animals and wildlife live in close proximity with the transfer of disease between them being of growing concern (Simpson et al. 2018). Gadaga et al. (2016) and Ndengu et al. (2017a) showed that livestock abortion is a huge challenge and farmers in these areas lack knowledge on possible abortion causes and their transmission pathways and are often at risk of contracting zoonotic infections because of risky animal husbandry practices and poor food handling of animal origin. Brucellosis has been serologically demonstrated in several herbivore wildlife species in the area (Caron et al. 2013; Gomo et al. 2012a; Madsen & Anderson 1995; Ndengu et al. 2017b) and cattle (Gomo et al. 2012a, 2012b; Ndengu et al. 2017b). Hence, because of the increased human-domestic animals-wildlife contacts in these areas and reliance of the communities on animals and their products for food, it is necessary to investigate goat brucellosis and chlamydiosis in order to highlight the associated public and animal health risks. The results of such studies may help in the design of appropriate control programmes for both animal and human brucellosis and chlamydiosis. In Zimbabwe, there have been limited studies on goat brucellosis (Halliwell, Honhold & Schlund 1987; Musarandoga & Muza 2013). To our knowledge, no studies have been conducted previously on goat chlamydiosis in Zimbabwe. Hence, the effects of these diseases on goats, wildlife and humans are largely unknown.

This study therefore seeks to investigate the presence and risk factors of brucellosis and chlamydiosis in goats at the livestock–wildlife interface area. The study aimed at establishing if proximity to wildlife is a risk factor for goat brucellosis and chlamydiosis by comparing their seroprevalences in communities representing three distinct areas: porous (unrestricted) and non-porous (restricted by fencing) livestock–wildlife interfaces and non-interface (absent) areas.

## Materials and methods

### Study location

The study was conducted in the south-eastern lowveld of Zimbabwe, a semi-arid area with annual rainfall below 500–600 mm. The study area falls under agro-ecological region V, a semi-arid land not suitable for rain-fed crop production. It is an extensive farming region with very low and erratic rainfall and therefore generally less suitable for grain and fodder crops. Farming depends on the utilisation of rangelands with extensive livestock production being most appropriate. The estimated goat population in the study area is about 150 000 goats and is composed mainly of the indigenous East African breed. Sheep were not considered in this survey as they are an uncommon domestic ruminant in the study area.

**FIGURE 1:**
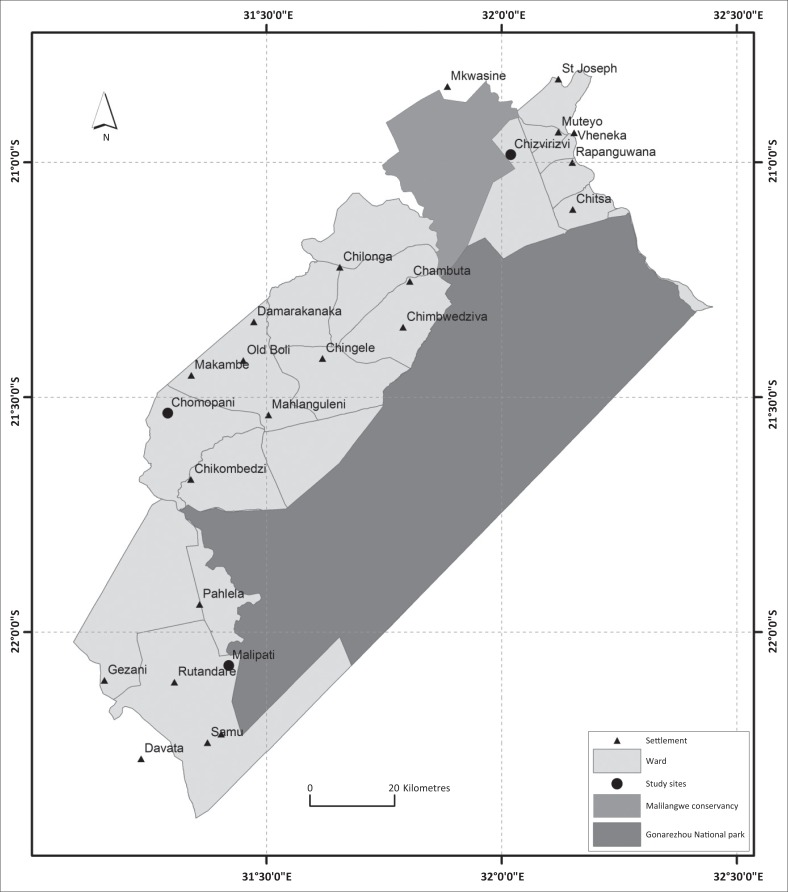
Map of the south-eastern Lowveld of Zimbabwe showing the Gonarezhou National Park and the adjacent Malilangwe Conservancy. The three studies are sites represented by big black dots.

Study areas were conveniently selected to include those with a livestock–wildlife interface in the Gonarezhou National Park (GNP) and Malilangwe Conservancy (MC) and another without a livestock–wildlife interface (Figure 1). The livestock–wildlife interfaces were a porous interface with unrestricted livestock–wildlife contact and a non-porous one with a fence preventing direct livestock–wildlife contacts (Ndengu et al. 2017a). The selected porous interface was Malipati rural village, which lies directly adjacent to the GNP boundary. A veterinary fence, erected in 1985 to prevent contacts between buffaloes and cattle and foot-and-mouth disease transmission, has been extensively damaged (Dube et al. 2010) permitting wildlife access to human settlements in Malipati and free movement of livestock into the GNP (Chigwenhese et al. 2016). Livestock share grazing and watering sources with wildlife, especially during the dry season when these resources are limited in the Malipati rural village (Miguel et al. 2013; Zengeya et al. 2015). Hence, a significant number of contacts between humans, livestock (including goats) and wildlife are assumed to occur at this interface type, potentially leading to pathogen transmission.

The selected non-porous interface was the Chizvirizvi village which lies on the periphery of the MC. Malilangwe Conservancy is located on the northern boundary of the GNP and it is surrounded by a well-maintained fence, preventing direct contact of wildlife in the conservancy with livestock and humans in the village. The conservancy has the full range of African wild ungulates occurring in the area, while the Chizvirizvi village hosts livestock, mainly cattle and goats. The fence creates a physically defined linear interface, separating wildlife and livestock (Ndengu et al. 2017a). The selected non-interface site was the Chomupani rural area which is located at least 15 km from the north-western boundary of the GNP. Wild ungulates are reportedly absent in this area and the site was considered to be a control site with no livestock–wildlife interactions as it was far away from the GNP (Ndengu et al. 2017a). Except for proximity to wildlife, all other factors including animal management practices and climatic conditions were similar for the selected study sites.

### Goat sampling and sample collection

In rural communities, a dip tank is a functional unit where disease surveillance and control activities are undertaken. A list of all farmers (representing households), who dip their cattle or those who are likely to use (without cattle) any of the dip tanks, is kept at the local veterinary office based in each of the areas (Ndengu et al. 2017a), this was taken as a representative household sampling frame. One dip tank was chosen for each selected study site based on its appropriate location to the interface and these were the Malipati (porous), Chizvirizvi (non-porous) and Chomupani (non-interface) dip tanks. The total number of farmers (households) listed from the three listed dip tanks was 2028 (Malipati = 697, Chomupani = 681 and Chizvirizvi = 650). Based on available resources, 25 households (representing flocks) per site, representing 3.7% of the total, were selected by a simple random procedure.

Goat blood samples were collected during the period September 2014–March 2016. Goats at each selected household were identified and sampled using a simple random method. The minimum goat sample size to be sampled was calculated using the formula:

$$n=\left[z^{2}\times p(1-P)\right]/e^{2},$$ [Eqn 1]

where

*z* is the value from standard normal distribution corresponding to the desired confidence level (*z* = 1.96 for 95% confidence interval), *p* is the estimated prevalence and *e* is the desired precision (Dohoo, Martin & Stryhn 2003). We estimated a prevalence of 50% for brucellosis or chlamydiosis and a 5% error margin at 95% confidence level. A minimum of 128 goats was therefore targeted per study site. Blood collection was performed by jugular venipuncture as suggested by Muma et al. (2006). Briefly, each selected goat was physically restrained and 15–20 mL of blood was collected by jugular venipuncture using a 20 mL disposable plastic syringe and an 18 gauge needle. The blood was immediately transferred to 4 mL plain tubes. All blood samples were left to stand for approximately 15 min in the shade at ambient temperature (2530 °C – 30 °C) to permit clot separation. Clotted blood samples were then centrifuged at 3000 *g* for 15 minutes and 2 mL of serum were collected into cryo-tubes and stored in liquid nitrogen at -196 °C *en route* to the laboratory. The cold chain was maintained during transportation of samples to the Faculty of Veterinary Science, University of Zimbabwe, and stored at -20 °C until analysis. During sample collection, epidemiological data pertaining to each individual were simultaneously collected using a questionnaire. The data included interface type, sex, flock size, reproductive failure history (abortions, still births and weak kids) and keeping of goats with cattle. Laboratory testing of the samples was performed at a World Organization for Animal Health (OIE) reference laboratory, Istituo Zooprofilattico Sperimentale of Abruzzo and Molise (IZSAM), Italy.

### Brucellosis testing

Antibodies to *Brucella* spp. were tested by using the Rose Bengal plate test (RBPT) and the complement fixation test (CFT). The tests were performed according to standard procedures as detailed in the Manual of Diagnostic Tests and Vaccines for Terrestrial Animals, OIE (2016). Antigens derived from strain 99 *B. abortus* biovar 1 (AHVLA, Weybridge, UK) were used. The positive and negative antigen reference controls were obtained from IZSAM, Teramo, Italy. The cut-off value for the CFT was 50% of haemolysis (++) at the 1:4 dilution, corresponding to 20 International Units (IU/mL). In this study, a serial testing protocol was used, and thus, a serum was considered positive for antibodies to *Brucella* spp. if it was positive to both the RBPT and the CFT.

### Chlamydiosis testing

The antibodies to *C. abortus* were detected by using the CFT. The test was performed using antigen and reference sera provided by IZSAM, Teramo, Italy. The sera were heat inactivated for 30 min and then diluted in a twofold series to cover a dilution range from 1/16 to 1/512. A total of 25 *µ*L of diluted sera, equivalent volume of antigen diluted according to the manufacturer’s (IZSAM) instructions and 2U complement were added to the plate wells and incubated at 37 °C for 30 min. After incubation, 25 *µ*L of 2% sheep red blood cells (SRBCs) sensitised with an equal volume of rabbit anti-SRBC serum diluted to contain 2U were added and the plate, after further incubation at 37 °C for 30 min, was centrifuged for 4 min at 1500 *g*. Positive and negative reference controls were included in the test. Samples with at least 100% fixation at the first dilution were considered positive; sera showing less than 100% fixation as the first dilution were considered negative.

### Data analysis

#### Descriptive statistics

The recording and editing of the data was performed in Microsoft Excel^®^ and the descriptive statistics were performed using Stata Version *SE* 10 for Windows (Stata Corp., College Station, TX, United States [US]). Animal husbandry practices in the study areas allow animals (cattle, goats) to be communally reared with common grazing and water sources. Thus, individual animal seroprevalence estimates with 95% confidence intervals (CIs) were computed using the Survey Command in Stata, with adjustment for study sites (porous, non-porous and non-interface) and sampling units (flocks). The overall number of brucellosis and chlamydiosis seropositive goats was calculated from the total number of samples tested over the study period and expressed as a percentage. A flock was considered to be positive if at least one goat tested positive for brucellosis or chlamydiosis. Seropositivity was examined in relation to epidemiological data collected. Interface type, sex, flock size, keeping goats with cattle and reproductive failure categories were generated as follows: three for interface type (porous, non-porous and non-interface), two for sex (female goats and male goats), two for flock size (≤ 20 and > 20), two for keeping goats with cattle (yes and no) and two for reproductive failure history (present and absent). The Chi-square test was used to measure differences between categories and values of *p* < 0.05 were considered as significant, while the odds ratio (OR) was used to evaluate the association between seropositivity and the epidemiological variables.

#### Multivariable logistic regression analyses

For female goats, a Survey Data Analysis in Stata was used to perform multivariable logistic regression analysis according to Dohoo et al. (2003). The analyses were performed using only data for female goats with complete records (*n* = 431); seven female goats had no data on abortion history. The dependent variable was the chlamydial seropositive status of goats (0 = negative, 1 = positive), while independent variables were the abortion history (0 = no, 1 = yes) and the interface type (porous, non-porous and non-interface). The OR was used to evaluate the association between seropositivity and the epidemiological variables

### Ethical considerations

Ethical approval for the use of goats and for all protocols used in this study was obtained from the Higher Degrees and Ethical committees of the Faculty of Veterinary Science, University of Zimbabwe and the Department of Veterinary Services, Zimbabwe. The purpose of this study was well explained to all the owners of the goats, who all expressed consent to participate in the study. Standard operating procedures were followed for collection of blood samples (Muma et al. 2006).

## Results

A total of 599 goat samples were collected with 38.1% (228) from Malipati (porous interface), 32.7% (196) from Chomupani (non-interface) and 29.2% (175) from Chizvirizvi (non-porous interface). Of the samples collected, 78.3% (469/599) were from female goats and 21.7% (130/599) were from male goats. However, 36 samples (1 for Chizvirizvi, 11 for Chomupani and 24 for Malipati) were not tested because of unsuitability or insufficient volumes, resulting in 563 samples being tested and analysed.

### Brucellosis seroprevalence

All of the goat sera tested (*n* = 563) were seronegative for *Brucella* spp. antibodies.

### Chlamydiosis seroprevalence

#### Univariable analysis

The overall individual animal-level seroprevalence was 22% and it varied significantly (*p* < 0.05) according to interface type (Table 1). At the flock level, the overall seroprevalence was 38.7% (29/75). Male goats had a higher seroprevalence but the difference was not significant (*p* = 0.5) (Table 1). Except for the non-interface site where chlamydial seroprevalence was significantly (*p* = 0.004) higher in female goats with an abortion history, no significant (*p* > 0.05) difference was noted for the other two sites, and overall, the difference was not significant (Table 2). Out of the 75 flocks, 48 (64%) reported cases of reproductive failure (abortions, still births, weak kids and neonatal deaths).

**TABLE 1:** Distribution of chlamydiosis seroprevalence according to interface and sex.

Category	Level	No. tested	Positive	Seroprevalence*	95% CI
-	All animals	563	124	22.0	18.7–25.7
Interface†	Porous (Malipati)	204	43	21.1^a^	11.4–30.7
	Non-porous (Chizvirizvi)	174	64	36.8^b^	22.8–50.7
	Non-interface (Chomupani)	185	17	9.2^c^	5.5–12.9
Sex	Female	438	93	21.2^a^	15.7–26.7
	Male	125	31	24.8^a^	14.4–35.2

CI, confidence interval.

* , Figures with a different superscript in the same column for the same category are significantly different at *p* < 0.05.

† , Porous interface: fence separating the site from park (Gonarezhou National Park) extensively damaged; Non-porous interface: an intact fence separating the site from game park (Malilangwe); non-interface: site far away from the boundary of the park (Gonarezhou National Park).

**TABLE 2:** Distribution of chlamydiosis seroprevalence in female goats according to abortion history.

Variable	Level	Porous		Non-porous		Non-interface		Overall	
		Tested	% positive (95% CI)	Tested	% positive (95% CI)	Tested	% positive (95% CI)	Tested	% positive (95% CI)
Abortion history†	Yes	28	32.1^a^ (16.6–52.4)	65	27.7^a^ (17.7–40.4)	67	16.4^a^ (8.9–27.9)	160	23.8^a^ (17.5–31.2)
	No	135	17.8^a^ (11.9–25.5)	64	45.3^a^ (33.0–58.2)	72	1.4^b^ (0.1–8.5)	271	19.9^a^ (15.4–25.3)

CI, confidence interval.

Figures with a different superscript are significantly different at *p* < 0.05.

† .Abortion status was not given for seven animals (porous = 1, non-porous = 2, non-interface = 4).

On univariable analyses, the interface type was significantly associated with chlamydial seropositivity, while sex and abortion history were not. The non-porous interface was 2.2 times and 5.8 times more likely to be seropositive than the porous interface (*c*^2^ = 10.7, OR = 2.2, 1.4 < OR < 3.4, *p* = 0.0011) and the non-interface (*c*^2^ = 37.5, OR = 5.8, 3.2 < OR < 10.3, *p* < 0.00001), respectively. Similarly, the porous interface was 2.6 times more likely to be chlamydial seropositivity than the non-interface site (*c*^2^ = 9.6, OR = 2.6, 1.5 < OR < 4.8, *p* = 0.002). Chlamydial seroprevalence was not associated with sex (*c*^2^ = 0.5, OR = 1.2, 0.8 < OR < 2.0, *p* = 0.5) and abortion history in female goats (*c*^2^ = 0.7, OR = 1.3, 0.8 < OR < 2.0, *p* = 0.4). Similarly, at flock level, there was no association between chlamydiosis seroprevalence and a history of reproductive failure (*c*^2^ = 0.2, OR = 1.6, 0.4 < OR < 6.0, *p* = 0.7), keeping goats with cattle (*c*^2^ = 0.2, OR = 1.5, 0.5 < OR < 4.9, *p* = 0.7) and flock size (*c*^2^ = 0.03, OR = 1.4, 0.4 < OR < 5.1, *p* = 0.9).

Overall, the majority (61.3%) of seropositive goats had a titre of 1:16, 21% a titre of 1:32 and 17.8% of them recorded a titre of 1:64 or higher (Table 3). Seven and three seropositive goats from the non-porous interface had titres of 1:128 and 1:256, respectively. None of the seropositive goats from the porous interface and the non-interface areas recorded a titre of 1:256 (Table 3).

**TABLE 3:** Distribution of chlamydiosis seroprevalence according to interface and titre.

Interface	No. positive	Titre									
		1:16		1:32		1:64		1:128		1:256	
		*n*	%	*n*	%	*n*	%	*n*	%	*n*	%
Non-interface	17	12	70.6	2	11.8	2	11.8	1	5.9	0	0.0
Porous	43	29	67.4	10	23.3	2	4.7	2	4.7	0	0.0
Non-porous	64	35	54.7	14	21.9	5	7.8	7	10.9	3	4.7

**Total**	**124**	**76**	**61.3**	**26**	**21.0**	**9**	**7.3**	**10**	**8.1**	**3**	**2.4**

#### Multivariable logistics regression analysis

The multivariable logistic regression model revealed study sites to be independently associated with the chlamydia seroprevalence of female goats (Table 4). The odds of chlamydia seropositivity increased from Chomupani to Malipati and Chizvirizvi, which are non-interface, porous interface and non-porous interface areas, respectively. Goats from Chizvirizvi were approximately six times (OR = 6.1, 2.5 < OR < 14.6) more likely to be seropositive for chlamydiosis compared to those from Chomupani (Table 4). Abortion history was not associated with chlamydial seropositivity (Table 4).

**TABLE 4:** Survey multivariable logistic regression analysis of the distribution of chlamydiosis seroprevalence according to interface and abortion history of female goats.

Variable	Level	Multivariable logistic regression†		
		*P*	Odds ratio	95% CI
Interface type	Non-interface (Chomupani)	-	1.0	-
	Porous (Malipati)	0.03	2.9	1.2–7.5
	Non-porous (Chizvirizvi)	0.000	6.1	2.5–14.6
Abortion history	No	-	1.0	-
	Yes	0.57	1.2	0.6–2.5

CI, confidence interval.

† , Overall data for the model: *p* = 0.001, number of observations = 431.

## Discussion

The RBPT is recommended for brucellosis screening (Garin-Bastuji & Blasco 1997), while the CFT is used for its confirmation in small ruminants (Alton 1990; MacMillan 1990) and the two tests were sequentially used in this study. The seronegative results indicated the absence of *Brucella* spp. in the tested goats. Similar results were reported on goats (*n* = 353) tested earlier in another part of the country (Musarandoga & Muza 2013). Muma et al. (2006) and Simpson et al. (2018) also showed the absence of brucellosis in goats tested at respective livestock–wildlife interface areas of neighbouring Zambia (*n* = 280) and South Africa (*n* = 593). The absence of *Brucella* spp. seropositive goats could be that the sampled flocks or goats were naturally free from *Brucella* infection. However, previous studies in the study area serologically demonstrated brucellosis in cattle and wildlife (Caron et al. 2013; Gomo et al. 2012a, 2012b; Madsen & Anderson 1995; Ndengu et al. 2017b) and *B. abortus* was isolated (Gomo et al. 2012b). Elsewhere, in the country, *B. melitensis* was previously diagnosed (Halliwell et al. 1987) but the goats in which the disease was confirmed were believed to have been illegally translocated across the border from Mozambique (Madsen 1989). One of the isolates was later identified as *B. melitensis* biovar 1 (Matope et al. 2009). In South Africa, *B. melitensis* biovar 1 was confirmed in goats (Ribeiro et al. 1990) and *B. melitensis* biovars 2 and 3 in cattle (Kolo et al. 2018). Besides freedom from the disease, we currently do not have any explanation on the absence of brucellosis seropositive goats from those sampled in the studied areas.

The CFT is the most widely used and recommended procedure for the detection of *C. abortus* in small ruminants (OIE 2012). It was used during the present study as reported elsewhere (Al-Qudah et al. 2004; Musuka et al. 2001; Santos et al. 2012). The test is able to detect antibodies from natural infection and vaccination (OIE 2012). As goat chlamydiosis vaccination is not practised in the study areas, the results indicate natural exposure to chlamydial infection and high titres observed in some of the goats are likely to be an indication of acute infection at or around the time of sampling. This study provides the first serological evidence of *C. abortus* infection in goats in the study areas of Zimbabwe. False-positive results are known to occur because of antigenic cross-reactivity but this is considered to be relatively rare (OIE 2012). Samples with at least 100% fixation at the first dilution were considered positive including those at a titre of 1/16. A titre of 1/16 could be because of a low-grade infection with *C. abortus* but it could be non-specific for *C. abortus* (OIE 2012).

The observed significantly lower goat chlamydial seroprevalence of the non-interface site compared to that of the interface sites (porous and non-porous) suggests that proximity to wildlife is likely associated with an increased chlamydial seropositivity in goats. Serological evidence of chlamydial infection was demonstrated in buffaloes (*Syncerus caffer*) and impalas (*Aepyceros melampus*) from the GNP (Ndengu et al. 2018). Contact between livestock and wildlife occurs at the porous interface (Miguel et al. 2013; Zengeya et al. 2015), and hence, wildlife could probably be a source of chlamydial infection in goats and vice versa. Elsewhere, chlamydial seropositivity was found to be higher in wild ruminants residing at the edge of a park where contact with domestic goats and sheep were more likely than those inhabiting the central area (Cubero-Pablo et al. 2000). However, seropositive goats from the non-interface and those from the non-porous interface are unlikely to have contact with wildlife. Goats from the non-porous interface recorded the highest chlamydial seroprevalence and, in contrast to our findings, cattle at the porous interface recorded the lowest chlamydial seroprevalence (Ndengu et al. 2018). These authors suggested a probable independent chlamydial infection cycle in buffaloes; positive sera were recorded in buffalo herds where contact with domestic stock was unlikely. These findings suggest that an independent chlamydial infection cycle is also likely in domestic ruminants. More convincing results could have perhaps been obtained by using more sites for each interface type. Further studies focused on isolates and strain typing will therefore provide more insights on *Chlamydia* pathogen sharing between domestic and wild ruminants in the study areas.

Ndengu et al. (2018) reported a high seroprevalence of chlamydiosis in cattle in the same study areas and our present results showed a moderately high goat chlamydiosis seroprevalence. In Namibia, farm prevalences of 25% and 86% have been reported, while individual goat seroprevalences ranged from 2.4% to 54% (Appel et al. 1989; Samkange et al. 2010). Elsewhere, very high prevalences of 91.7% have been reported (Krkalic et al. 2015). The communal sharing of pastures and water sources and keeping together of domestic ruminants practised in the study areas could play a role in the observed high seroprevalence. In addition, the massive environmental contamination which occurs at abortion or parturition when infected female goats shed vast numbers of infective *C. abortus* (Rodolakis & Laroucau 2015) forms a major source of infection. The natural route of transmission is considered to be mostly by ingestion or inhalation of infected materials, for instance, when grazing in contaminated pastures (Rodolakis & Laroucau 2015). Hence, the animal husbandry practices in conjunction with high environmental contamination and various transmission routes are likely to provide a higher risk of chlamydial exposure to domestic ruminants in the study areas.

As was observed elsewhere (Zhao et al. 2012), the risk of chlamydial seropositivity was independent of goat sex. The seroprevalence of *C. abortus* was previously reported to be independent of goat flock size (Al-Qudah et al. 2004; Santos et al. 2012) and this is consistent with our findings. Our results also suggest that owning multiple domestic ruminants is not associated with an increased risk of chlamydiosis seropositivity. The open grazing system practised in the study areas allows mixing of flocks or herds and interspecies grazing; this likely contributes to a uniform spread of chlamydiosis and other infections thereby confounding the effects of flock size and multiple domestic ruminant ownership (Ndengu et al. 2017a). However, the dichotomisation of flock size may result in loss of information and sometimes even inaccurate results (Collins et al. 2016).

Chlamydial infection in goats is known to be associated with abortions (Aitken & Longbottom 2007; Matthews 1999; Musuka et al. 2001; Nietfeld 2001; Rodolakis & Laroucau 2015). Abortion history was shown to be associated with a higher risk of chlamydial seropositivity in goats (Samkange et al. 2010; Santos et al. 2012). This is contrary to our observations where history of abortions and other reproductive problems were found not to be associated with chlamydial seropositivity. Similar results were recorded in cattle from the same study areas (Ndengu et al. 2018). In Sardinia (Italy), Masala et al. (2005) reported that *C. abortus* had a relatively minor role in caprine and ovine abortion. Goats and ewes usually abort once (Rodolakis & Souriau 1980) and after abortion, the affected animals are immune and are unaffected in subsequent pregnancies (Nietfeld 2001). Data collected on abortion history were restricted to its previous presence or absence and were not specific on whether abortion occurred in the first or subsequent pregnancies. No production and reproduction records were kept by the sampled farmers and data collected relied on recall by the farmers. Hence, lack of accurate data on when abortions occurred could have probably affected the observed association. In addition, low titres of less than 1/32 are non-specific for *C. abortus* (OIE 2012) and this could also perhaps have biased our findings. The role of chlamydial infection on goat reproductive failure in the country needs further investigation utilising large samples from flocks where reproductive data are accurately captured.

In conclusion, the present study demonstrated the respective absence and presence of brucellosis and chlamydiosis in goats sampled in the south-eastern lowveld of Zimbabwe. Our results suggest that proximity to wildlife is likely associated with an increased chlamydial seropositivity in goats. More studies are required to determine the public health risk of chlamydiosis and the role of chlamydial infection on goat reproductive failure and that of wildlife on *C. abortus* transmission to goats and other domestic ruminants.
